# Trajectories of Self-Rated Health in People with Diabetes: Associations with Functioning in a Prospective Community Sample

**DOI:** 10.1371/journal.pone.0083088

**Published:** 2013-12-10

**Authors:** Norbert Schmitz, Geneviève Gariépy, Kimberley J. Smith, Ashok Malla, Richard Boyer, Irene Strychar, Alain Lesage, JianLi Wang

**Affiliations:** 1 Department of Psychiatry, McGill University, Montreal, Quebec, Canada; 2 Douglas Mental Health University Institute, Montreal, Quebec, Canada; 3 Department of Epidemiology and Biostatistics, McGill University, Montreal, Quebec, Canada; 4 Montreal Diabetes Research Centre, Montreal, Quebec, Canada; 5 Centre de Recherche Fernand Seguin, Hôpital Louis-H. Lafontaine, University of Montreal, Montreal, Quebec, Canada; 6 Departments of Psychiatry and Community Health Sciences, Faculty of Medicine, University of Calgary, Calgary, Canada; 7 Department of Nutrition, Faculty of Medicine, University of Montreal, and the Centre de Recherche du Centre Hospitalier de l’Université de Montréal (CRCHUM), Montreal, Quebec, Canada; Iran University of Medical Sciences, Iran (Islamic Republic Of)

## Abstract

**Background:**

Self-rated health (SRH) is a single-item measure that is one of the most widely used measures of general health in population health research. Relatively little is known about changes and the trajectories of SRH in people with chronic medical conditions. The aims of the present study were to identify and describe longitudinal trajectories of self-rated health (SRH) status in people with diabetes.

**Methods:**

A prospective community study was carried out between 2008 and 2011. SRH was assessed at baseline and yearly at follow-ups (n=1288). Analysis was carried out through trajectory modeling. The trajectory groups were subsequently compared at 4 years follow-up with respect to functioning.

**Results:**

Four distinct trajectories of SRH were identified: 1) 72.2% of the participants were assigned to a persistently good SRH trajectory; 2) 10.1% were assigned to a persistently poor SRH trajectory; 3) mean SRH scores changed from good to poor for one group (7.3%); while 4) mean SRH scores changed from poor to medium/good for another group (10.4%). Those with a persistently poor perception of health status were at higher risk for poor functioning at 4 years follow-up than those whose SRH scores decreased from good to poor.

**Conclusions:**

SRH is an important predictor for poor functioning in diabetes, but the trajectory of SRH seems to be even more important. Health professionals should pay attention to not only SRH per se, but also changes in SRH over time.

## Introduction

Diabetes is a chronic condition associated with reduced physical functioning [1]. There is increasing interest in using brief health status measures to monitor health changes and health service needs in people with diabetes. Those measures provide important information on population health over time and enables informed decisions to be made about the implementation and evaluation of public health action. Furthermore, those measures can be used for the measurement of disparities within countries and comparisons across countries [2]. 

Self-rated health (SRH) is a single-item measure that is one of the most widely used measures of general health in population health research. The most commonly used form asks people to characterize their health as excellent, very good, good, fair, or poor. SRH has received strong support as an independent predictor of morbidity, disability, health service utilization and mortality [3,4], even when adjusted for objective health indicators. It has been recommended for use in health monitoring by the World Health Organization, the US Centers for Disease Control, the Institute of Medicine in the United States and the European Commission [5-8]. Despite its extensive use as a brief health status measure with consistent predictive power in cohort studies, it is not clear what SRH exactly measures and why it has such strong associations with morbidity and mortality. There is increasing evidence that biological, physiological, and psychological factors are major determinants of SRH [9]. Self-rated health may reflect a subjective summary of all the information people have available about their understanding of health, including information on physical functioning in everyday life, lifestyle conditions that have adverse effects on health, information from health professionals, specific disease characteristics, mood and personality factors [9]. Therefore, SRH might be a dynamic evaluation of health, incorporating past health experience with current health conditions and future health expectations [10,11]. Using data from a large American community sample, Zajacova et al. [12] found that a substantial proportion of individuals (40%) changed their ratings of health across two interviews (one month apart). Han et al. [11] reported that a decline in SRH was a stronger predictor for mortality than a single measurement occasion in a sample of older women. They concluded that the rating of current health status is important but that the way participants arrive at their current health state might be even more important.

Although it is well known that SRH is a dynamic perception of health status that changes over time [13], relatively little is known about changes and the trajectories of SRH in people with chronic medical conditions. Most of the prospective studies have focused on general population samples [14,15]. These studies have identified different patterns of SRH over time; for example, persistently good health, good but declining health, persistently fair health, and fair but declining health [16]. The temporal change of SRH might be different for people with diabetes due to the progress and management of the condition. 

The aims of the present paper were to: (1) determine whether a set of SRH trajectories can be identified in a community sample of people with diabetes; (2) describe sociodemographic and clinical characteristics associated with these trajectories; and (3) examine the effect of different trajectories of SRH on functioning outcome. 

Identification of trajectory groups of SRH may provide a deeper understanding of SRH as a risk factor for variation in health outcomes in people with diabetes and might help identifying high risk groups in this population. 

## Materials and Methods

### Ethics Statement

The Douglas Mental Health University Institute Ethics Board approved the consent procedures and the study protocol. All participants gave oral informed consent prior to their inclusion in the study. Oral consent was documented by the interviewer.

### Study design and participants

We used data from the longitudinal Montreal Diabetes Health and Well Being Study (DHS). The DHS is a community based telephone survey of adults with self-reported diabetes in Quebec, Canada. Participants were recruited by a recognized polling firm (Bureau d'intervieweurs professionnels, Montreal, QC) between January 2008 and April 2008 through random selection of phone numbers. Interviews were conducted in English and French by trained professional interviewers using a computer-assisted telephone interview system (86,486 phone calls were made, 62,439 persons were reached, 54,930 persons accepted to be interviewed, 3,221 persons were eligible for the interview, and 2,003 persons completed the interview).The sampling frame consisted of all households with a listed telephone number in Quebec, Canada. Eligible participants were individuals who were between 18 and 80 years of age and had a diagnosis of type 1 or type 2 diabetes. Four follow-up interviews were conducted approximately 12, 24, 36 and 48 months after baseline interview (late winter/early spring). More details from the baseline assessment are published elsewhere [17]. 

### Measures

SRH was assessed with one item: “Would you say that in general your health is…” Participants were asked to answer with a 5-category Likert response scale (excellent, very good, good, fair or poor). We used the coding suggested by Diehr et al. [18], with 95 for excellent, 90 for very good, 80 for good, 30 for fair, 15 for poor, and 0 for decedents. This coding scheme was developed from large longitudinal studies of older adults and reflects the estimated percentage probability of persons being healthy (defined as being in excellent, very good, or good health) two years later, conditional on the current observed value. The coding incorporates the decedents in the trajectory estimation after their deaths [16]. These scores can be thought of as a global measure of health, where 0 is death and 100 is perfect health. 

Global functioning was assessed using the 12-item version of the World Health Organization Disability Assessment Schedule II (WHO-DAS-II) [19]. The WHO-DAS-II assesses functioning during the past 30 days in domains defined by the WHO International Classification of Functioning, Disability and Health (ICF), including self-care, mobility, understanding and communication, interpersonal relations, work and domestic responsibilities and participation in community activities. Several studies have shown that the WHO-DAS-II is a reliable instrument for the assessment of disability in people with chronic conditions [20]. The WHO-DAS-II summary score was transformed to percent score (0% to 100%), with higher scores reflecting greater disability. Based on available normative data, Andrews et al. suggested that a WHO-DAS-II score of 21 or greater indicates clinically significant level of poor functioning [21]. 

The Patient Health Questionnaire (PHQ-9) [22-24] was used to assess depressive symptoms. Participants were asked to what extent they had experienced nine depressive symptoms over the past two weeks. A classification into minor or major depression was used, based on an algorithm derived from diagnostic criteria of the Diagnostic and Statistical Manual of Mental Disorders, Fourth Edition [22]. The PHQ-9 is widely accepted as a valid measure of depression severity in medical settings [25]. The internal consistency of the PHQ-9 was 0.86 in a recent study of people with diabetes [26]. The DHS collected data on socio-demographic characteristics, including age, sex, marital status and educational level. Participants were asked whether they currently smoked, whether they ever smoked and to rate the number of days they exercised or participated in sports for at least 15 minutes in the last month. The latter was collapsed into two categories: (0 days: inactive; >0 days: active). Body mass index (BMI) was calculated based on self-reported weight and height (weight in kilograms divided by the square of height in meters). Participants were asked whether they suffered from various chronic health conditions (asthma, high blood pressure, heart disease, stomach or intestinal ulcers, arthritis or rheumatism, migraine headaches, cancer, kidney disease, and back problems). Diabetes complications were assessed using the 17-item Diabetes Complications Index (retinopathy, neuropathy, large-vessel atherosclerotic disease, peripheral vascular disease, cerebrovascular disease and foot problems) [27]. Duration of diabetes was calculated based on the age at which participants were first diagnosed with diabetes. 

Participants with age at diagnosis < 30 years and insulin use immediately after diagnosis were epidemiologically classified as having type 1 diabetes, while other participants were classified as having type 2 diabetes. 

### Statistical analysis

To identify distinct trajectories of self-reported health (baseline, one-, two- and three-years follow-up assessments), we used trajectory modeling [28,29]. We used ‘proc traj’, a SAS macro which fits a semiparametric mixture model to longitudinal data with the use of the maximum-likelihood method [28,29]. Because transformed SRH scores could not be higher than 95 (for excellent health) and lower than 0 (for death), a censored normal distribution approach was used, with the likelihood function including the probability of observing scores of 95 (ceiling effects) and 0 (floor effects). To select the number of trajectory groups, we considered several factors: the Bayesian Information Criterion (BIC); statistical significance of quadratic and cubic terms of time and membership probabilities. We used the BIC to test from two to seven trajectories. We first assessed if a model with an additional group was a better ‘fit’, based on the criteria listed above and membership probabilities, and then assessed for the model specification (linear, quadratic and cubic terms) that best defined the trajectory in each group. We started with a cubic specification for trajectory shape, and dropped non-significant polynomial terms. These analyses were repeated after adjustment for age, sex and education. Each individual was assigned to the trajectory group for which he/she had the highest posterior probability of membership (maximum probability assignment rule)[30]. We aimed for groups with membership probabilities of at least 5% [30]. Within each group, values of average posterior probability of group membership were computed for each trajectory identified in the data. This probability is an approximation of the internal reliability for each trajectory.

In the ‘proc traj’ macro, subjects do not need complete data for all four assessment points to be included in the analysis. Missing data are assumed to be missing at random. 

We compared prevalence of sociodemographic variables and baseline health characteristics by trajectory group (Chi-square tests and general linear models were used for categorical and continuous variables, respectively).

The derived trajectory groups (from baseline to three years follow-up) were subsequently compared at 4 years follow-up with respect to functioning. Logistic regression analysis was conducted to compare the risk of poor functioning for the different trajectories while controlling for potentially confounding variables, including poor functioning history, depression history, complications history, type of diabetes, duration of diabetes, age, sex, and education. Contrasts were used to determine whether disability differed between the trajectories groups.

## Results

A total of 2003 individuals with type 1 or type 2-diabetes participated in the DHS at baseline (53% female). After excluding those who refused to participate in a follow-up interview (n=246), 1757 individuals formed the baseline sample for the longitudinal cohort. The number of participants with complete information on SRH at baseline and the three follow up interviews was 1746, 1287, 1142 and 1144, respectively. The number of deceased participants at the three follow-up interviews was 32, 17 and 12, respectively. For the trajectory analysis, we included participants who a) had complete information on SRH at baseline and b) participated in at least two follow up interviews or were deceased. A total of 1288 people had complete information on SRH in at least two follow-up assessments and 948 of those participants participated in the four years follow-up assessment.

The participants and those who dropped out did not differ in SRH scores at baseline and follow-ups. Participants who dropped out had poorer functioning status (p<0.001), more diabetes specific complications (p<0.001) and suffered more often from depressive symptoms (p<0.001) at baseline than those who did not drop out. 

Mean SRH scores decreased minimally over time: the scores for baseline and the three follow-up assessments were 71.8 (SD=24.2), 71.2 (SD=25.8), 68.6 (SD=27.8) and 68.5 (SD=28.4), respectively. 

**Figure 1:**
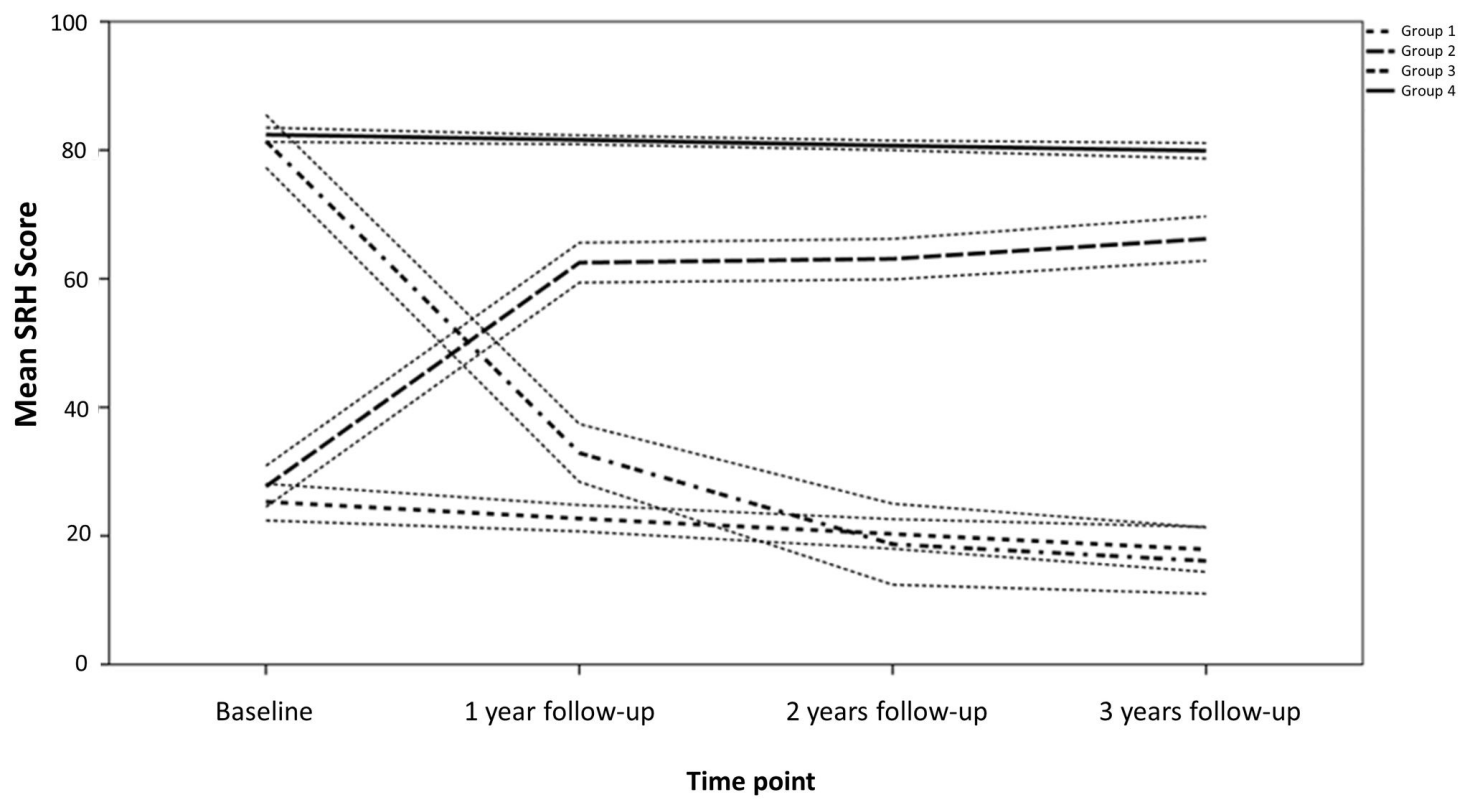
Self Rated Health (SRH) Trajectories, with 95% Confidence Intervals, for the Four Group Model (with Age, Sex and Education as Covariates), in the Montreal Diabetes Health and Well Being Study, 2008-2011. Note. Group 1: persistently poor SRH; Group 2: progressively increased SRH; Group 3: progressively decreased SRH; Group 4 persistently good SRH.

Four distinct trajectories of SRH were identified: (1) persistently poor self-rated health (P-SRH; 10.1 %); (2) progressively increased self-rated health (I-SRH; 10.4%); (3) progressively decreased self-rated health (D-SRH; 7.3 %); and (4) persistently good self-rated health (G-SRH; 72.2 %). The results of the trajectory analysis are presented in Table 1. The four identified SRH group trajectories are shown in Figure 1. SRH scores for each survey year were calculated from the beta coefficients presented in Table 1. SRH scores of group 1 (P-SRH) and group 2 (G-SRH) did not change much over time: there was a small linear decrease of SRH scores over time. In contrast, members of the D-SRH group had high SRH scores at baseline, similar to the G-SRH group and low SRH after three years, similar to the P-SRH group. Members classified as belonging to the I-SRH group had low SRH scores at baseline and medium to good SRH scores after three years. 

**Table 1:** Estimated Regression Parameters from the Trajectory Modeling in the Montreal Diabetes Health and Well Being Study, 2008-2011.

	Intercept (SE)	Linear term (SE)	Quadratic term (SE)	Cubic term (SE)
Group 1 Persistently poor SRH	25.01 (1.15)	-2.65 (0.64)	-	-
Group 2 progressively increased SRH	27.54 (1.35)	64.56 (5.48)	-35.75 (4.83)	6.18 (1.06)
Group 3 progressively decreased SRH	82.18 (1.54)	-74.07 (7.65)	28.49 (7.12)	-3.75 (1.59)
Group 4 persistently good SRH	83.32 (0.42)	-0.94 (0.23)	-	-

The mean posterior probability value of each group in the four-group model was 0.96 or higher. 

Sociodemographic and clinical baseline characteristics are presented in Table 2. There were important differences between the four trajectory groups with respect to sociodemographic and clinical characteristics. Participants in the G-SRH group had a higher educational level, better lifestyle-related behaviors (lower rates of smoking, physical inactivity and obesity) and better functioning (lower rates of disability, fewer chronic conditions and lower rates of minor or major depression) at baseline compared to the other three groups. Those in the P-SRH group had more diabetes-specific complications, higher rates of poor functioning, higher rates of depressive symptoms, were more often smoker and more often physically inactive than participants in the other three groups. The I-SRH had poorer functioning and poorer lifestyle-related behaviors compared to the D-SRH group at baseline. 

**Table 2:** Baseline Characteristics of the Four Trajectory Groups in the Montreal Diabetes Health and Well Being Study, 2008-2011.

Baseline	Group 1 persistently poor SRH (n=128)	Group 2 progressively increased SRH (n=134)	Group 3 progressively decreased SRH (n=92)	Group 4 persistently good SRH (n=919)	p-value
Sex, %					0.029
Women	60.9	63.4	50.0	52.2	
Age, M (SD)	60.2 (10.7)	57.9 (10.6)	62.8 (10.8)	57.8 (11.9)	<0.001
Education, %					0.021
< secondary school	45.3	49.3	46.7	38.4	
secondary school	31.3	22.4	19.6	28.4	
> secondary school	23.4	28.3	33.7	33.2	
Marital Status, %					0.002
Married/Living as married	50.4	56.7	56.0	66.3	
Divorced/Separated/Widowed	33.9	29.9	34.1	21.7	
Single	15.8	13.4	9.9	12.0	
Ethnicity, %					
White/Caucasian	91.3	91.8	89.0	93.6	0.336
Diabetes, %					0.062
Type 2	94.5	96.3	97.8	92.1	
Diabetes Duration, M (SD)	14.2 (11.9)	15.2 (13.3)	12.1 (11.2)	11.0 (10.8)	<0.001
Insulin use, %					<0.001
Yes	46.1	34.3	20.7	26.5	
Diabetes specific complicat., %					<0.001
0	7.8	16.7	16.7	38.5	
1	15.7	23.0	32.1	30.5	
2 and more	76.5	60.3	51.2	31.0	
BMI, %					<0.001
Overweight	20.2	22.0	33.7	37.7	
Obese	59.7	57.2	46.5	39.2	
Smoking, %					0.001
Current smoker	31.3	20.9	22.8	17.3	
Former smoker	44.5	41.8	50.0	43.6	
Physical activity, %					<0.001
Inactive	56.8	41.7	33.7	20.6	
Poor Functioning, %					<0.001
WHO-DAS-II >20	71.1	50.8	20.7	11.5	
Chronic conditions, %					<0.001
0	4.2	8.7	15.7	24.2	
1	8.5	18.1	33.7	30.6	
2 and more	87.3	73.2	50.6	45.2	
Minor or major depression,%					<0.001
PHQ-9	54.7	33.6	22.0	10.6	

At three years follow-up assessment, 79.3% of participants in the P-SRH group had a history of depressive symptoms (baseline to three years follow up). Rates of depression history for the I-SRH, D-SRH and G-SRH groups were 57.3%, 67.4 %, and 27.9 %, respectively. Rates of poor functioning history were 86.7%, 68.9%, 82.6% and 24.4% for participants in the P-SRH, I-SRH, D-SRH and G-SRH groups, respectively.

Table 3 presents the results of the logistic regression for the association between poor functioning at 4 years follow-up and SRH trajectories. After adjusting for potentially confounding factors (poor functioning history, depression history, type of diabetes, duration of diabetes, age sex and education), the P-SRH trajectory was associated with a six times increased odds of poor functioning after 4 years (OR=5.9) while both D-SRH and I-SRH trajectories were associated with a more than two times increased odds of poor functioning after 4 years (OR=2.3and OR=2.0, respectively) compared to the G-SRH trajectory. Both D-SRH and I-SRH groups had a lower odds of poor functioning at 4 years follow-up compared to the P-SRH group. 

**Table 3:** Results of the Logistic Regression Analysis for Predicting Poor Functioning in the Montreal Diabetes Health and Well Being Study, 2008-2012.

	Poor functioning at 4 years follow-up assessment, %	aOR	95 % CI	aOR (Group 1 Refer.)	95 % CI	aOR (Group 2 Refer.)	95 % CI
Group 1 persistently poor SRH	73.5	5.87	3.01, 11.42	1			
Group 2 progressively increased SRH	41.8	1.99	1.19, 3.31	0.33	0.16, 0.71	1	
Group 3 progressively decreased SRH	52.2	2.33	1.15, 4.72	0.40	0.17, 0.06	1.18, 1.17	0.54, 2.57
Group 4 persistently good SRH	13.5	1					

Note. 924 participants were included in the regression analyses.

Abbreviations: aOR, adjusted odds ratio; CI, confidence interval.

In sensitivity analyses we repeated our analyses for participants with complete SRH information at all three follow-up assessments. In these analyses, the number of groups and group characteristics did not differ systematically. 

## Discussion

In this prospective community study of people with diabetes, we have identified four distinct trajectories of SRH over a three-year period. The vast majority of the study participants had no major change of SRH status over the course of three years: 72 % were assigned to a persistently good SRH trajectory, while 10% were assigned to a persistently poor SRH trajectory. In contrast, perception of health status changed for the other two groups: one group changed from good to poor SRH, while the other group changed from poor to medium/good SRH. 

Our study adds to the current literature in three ways. First, we have evaluated short-term change of SRH in community sample of people with a chronic condition (diabetes), rather than evaluating long-term change of the SRH in a general population sample. Second, using trajectory modeling, we have identified one subgroup of participants with an improved self-rated perception over time. Most of the previous prospective studies on the course of SRH in community based samples have identified stable or decreasing trajectories of SRH [16,31-33]. Our results suggest that there might be additional (short-term) trajectories of SRH in people with chronic conditions like diabetes: for example, a trajectory where perception of health improves over time. The course of the chronic condition might affect health perception. For instance, changes in treatment or changes in lifestyle behaviors might improve diabetes management and reduce complications, which in turn might result in a better perception of health status. 

Third, we found that SRH history might be a better predictor for poor functioning than a single assessment of SRH. For example, the progressively decreased SRH group and the persistently poor SRH group had similar poor SRH scores at three years follow-up assessment, but the risk of poor functioning at four years follow-up was different: those with a persistent poor perception of health status were at higher risk for poor functioning than the other group where health perception changed over time, even after controlling for poor functioning history and other potentially confounding variables. Furthermore, the two groups with changing health perception over time - D-SRH and I-SRH - had different SRH scores at three years follow-up, but both groups had a similar risk for poor functioning at the 4-year follow up. 

SRH is a widely used measure; the usefulness of this single question is well established [9,34]. Nevertheless, it is not clear what exactly SRH measures. It is likely that it is an umbrella construct under which different dimensions such as physical, mental, social, and general well-being reside. Those dimensions may vary between individuals, where some may consider well-being as more important than symptoms.

The SRH measure is often dichotomized into two categories (fair/poor vs. excellent/very good/good) in epidemiological research. In the present study we used a coding suggested by Diehr et al. [18] which can be interpreted as the estimated probability of future health, or more generally as a global measure of health status. This coding takes into account that the response categories excellent, very good and good are similar but slightly different indicators for good health status, while the response categories fair and poor are similar but slightly different indicators for poor health status. The high stability of SRH observed in our study might be in part explained by this coding scheme: participants who switch from excellent to very good SRH or from very good to good SRH have only a minor change in SRH scores, while those who switched form excellent/very good/good to fair/poor SRH have a major change in SRH scores.

A limitation of this coding, as well as of the SRH measure, is the lack of medium health category. 

The role of SRH as a risk factor for poor health outcomes has been shown in the literature [35,36], although most studies have focused on mortality or disability pension in the general population. Idler and Benyamini [37] suggested that SRH might be a dynamic evaluation of health status, judging the trajectory of health and not only current health at a defined point in time. SRH might also reflect resources linked to one’s ability to cope with health threats (both external resources such as education, social support, access to care and internal resources such as optimism, vigour and perceived control). Our results support the idea that SRH is a dynamic evaluation of health status, but it seems to be a somewhat robust dynamic evaluation. SRH is associated with many aspects of health. Hence one might expect that SRH would fluctuate along with these aspects. But the present study suggests a high stability of SRH over time in people with diabetes, at least in a short time interval (3 years). It is likely that SRH is only sensitive to major changes in the different health dimensions and/or other factors not directly related to health. 

The study has limitations. SRH is a very brief measure and its longitudinal validity is threatened by ceiling effects (people in the highest category of SRH cannot improve). The PHQ-9 and the WHO-DAS-II are brief questionnaires for the assessment of depression and functioning. Functioning is complex, multi-dimensional phenomenon and a global self-report instrument might not cover all aspects of functioning. Furthermore, we have no information about functioning history before baseline and between follow-up assessments. We also have no information on clinical parameters like blood glucose levels. Another limitation is that our other study variables were self-reported and taken at one point in time, which may not fully capture a person’s life-time experiences. The sampling frame was limited to those with landline telephones, which might result in selection bias. Attrition might be another source of bias, although there was no evidence of different SRH scores in those lost to follow-up. Finally, our findings may not be valid for people with undiagnosed diabetes.

In conclusion, our results confirm that current SRH is an important predictor for disability in diabetes, but the trajectory of SRH seems to be even more important. Health professionals should pay attention not only to SRH per se, but also changes in SRH over time (e.g., decline of SRH from very good to fair or poor) among people with diabetes. A decline in SRH over time indicates that health status is deteriorating and that there is also an increased risk of poor functioning. 
